# Work ability and mental health among healthcare workers

**DOI:** 10.1192/j.eurpsy.2025.2014

**Published:** 2025-08-26

**Authors:** I. Sellami, A. Abbes, A. Feki, A. Haddar, H. Halweni, M. Hajjaji, S. Baklouti, M. L. Masmoudi, K. Jmal Hammami

**Affiliations:** 1 Occupationnal medecine; 2 Rheumatology, CHU Hedi Chaker; 3 General practice, Medecine university, Sfax, Tunisia

## Abstract

**Introduction:**

Mental health can affect the quality of life and productivity of healthcare workers (HCWs). It is important to determine the effect of mental health on work ability among these workers to implement adequate interventions.

**Objectives:**

Our study aims to assess the relationship between work ability and mental health among HCWs.

**Methods:**

We conducted a descriptive, analytical and cross-sectional survey among HCWs using a self-administrated questionnaire. We collected socio-professional data. We assessed work ability using the work ability index (WAI) and mental health using the depression anxiety and stress scale (DASS 21).

**Results:**

Our population comprised 200 HCWs, 71% of whom were female. The mean age of participants was 42.9 years. The mean of the tenure of job was 14.2 ±10.1 years. We found that 5.5% presented mild to moderate stress, 3% severe to extremely severe stress, 4.5% presented severe to extremely severe depression, 7.7% presented severe to extremely severe anxiety and 2.5% presented severe to extremely severe depression. Using the WAI, we found that 63% of participants perceived poor to moderate work ability and 37% perceived good to excellent work ability. Work ability was negatively correlated with stress (p = 0.005, r = -0.19), depression (p = 0.003, r = -0.2) and anxiety (p = 0.003, r = -0.2).

**Conclusions:**

Our findings highlight a correlation between HCWs’ altered mental health and poor work ability. Therefore, actions to promote mental health among these workers are urgently needed not only to improve the work ability and productivity of this population.

**Disclosure of Interest:**

None Declared

